# The chloroplast genome of silk floss tree (*Ceiba speciosa*)

**DOI:** 10.1080/23802359.2019.1677188

**Published:** 2019-10-18

**Authors:** Songyi Huang, Qiaoming Zhu, Guilian Huang, Bingqian Han, Qiujie Zhou, Jinhong Dai

**Affiliations:** a The Ecological & Landscape Research Center, Guangzhou Landscaping Company, Guangzhou, China;; b The General Manager Office, Guangzhou Minghui Landscape Technology Co. Ltd, Guangzhou, China;; c State Key Laboratory of Biocontrol and Guangdong Provincial Key Laboratory of Plant Resources, School of Life Sciences, Sun Yat-sen University, Guangzhou, China

**Keywords:** *Ceiba speciosa*, chloroplast genome, Malvaceae, illumina sequencing

## Abstract

*Ceiba speciosa* (Malvaceae), also called silk floss tree, is a beautiful and deciduous tree native to tropical and subtropical forests of South America. Its phylogenetic position remains unclear. In this study, the complete chloroplast genome sequence of *C. speciosa* was reported. Its chloroplast genome size was 160,360 bp, which contains a small single copy (SSC) region of 19,947 bp and a large single copy region (LSC) of 89,393 bp, and two inverted repeats (IRs) of 25,510 bp each. In total, 129 genes were annotated for the chloroplast genome, including 86 protein-coding genes, 37 tRNA genes and 8 rRNA genes. Phylogenetic analysis showed that *C. speciosa* was sister to *Bombax ceiba.*

*Ceiba speciosa*, also called silk floss tree, is a beautiful and deciduous tree native to the northeast of Argentina, east of Bolivia, Paraguay, Uruguay and southern Brazil (Gibbs and Semir 2003). It is an important landscaping tree widely cultivated in tropical and subtropical forest areas of the world, such as South Africa, South China and Southeast Asia. In this study, the chloroplast genome of *C. speciosa* was sequenced and characterised, to infer its phylogenetic position.

The fresh leaf tissue of *C. speciosa* was collected in the campus of Sun Yat-sen University (113°17′E, 23°5′N), Guangzhou, China. The voucher specimen (CS20180628) was stored in the Herbarium of Sun Yat-sen University (SYS). The total DNA was extracted with the Omega D5511-00 SP Plant DNA Kit. The DNA library was prepared with a TruSeq DNA Sample Prep Kit (Illumina, USA) according to the instructions of the manufacturer. Then the DNA library was sequenced on an Illunima Hiseq X Ten system at Vazyme Biotech Co. Ltd (Suzhou, China). A total of 7.5 Gb short read sequence data was generated and then utilised to assemble its chloroplast genome in NOVOPlasty (Dierckxsens et al. 2017) with the chloroplast sequence *rbcL* of *C. speciosa* (GenBank accession number: MG718425) as the seed. The genome was annotated on the online tool DOGMA (Wyman et al. 2004) with default parameters. For the phylogenetic analysis, the chloroplast genomes of *C. speciosa* and 9 other species were aligned using MAFFT (Katoh and Standley 2013). *Paeonia suffruticosa,* a species of Paeoniaceae was selected as the outgroup. A phylogenetic tree was constructed with maximum likelihood method by using RAxML (Stamatakis 2014).

The complete chloroplast genome of *C. speciosa* (GenBank accession number: MK820674) was 160,360 bp in length, with GC content of 35.x%. The chloroplast genome contains a large single-copy (LSC) region of 89,393 bp, a small single-copy (SSC) region of 19,947 bp, separated by a pair of inverted repeat region (IRs) of 25,510 bp. 129 genes were predicted in the whole chloroplast genome, including 37 tRNA, 8 rRNA, and 86 protein-coding genes.

The phylogenetic analysis showed *C. speciosa* was closest to *Bombax ceiba,* another species in Malvaceae (Figure 1). The two genera, *Ceiba* and *Bombax*, were previously placed in Bombacaceae (Watson 1992) and this family was recently recognised as subfamilies of Malvaceae (Heywood et al. 2007; Takhtajan 2009). Thus, our results support this treatment. The chloroplast genome of *C. speciosa* reported here provides new resources for further study.

**Figure 1.**
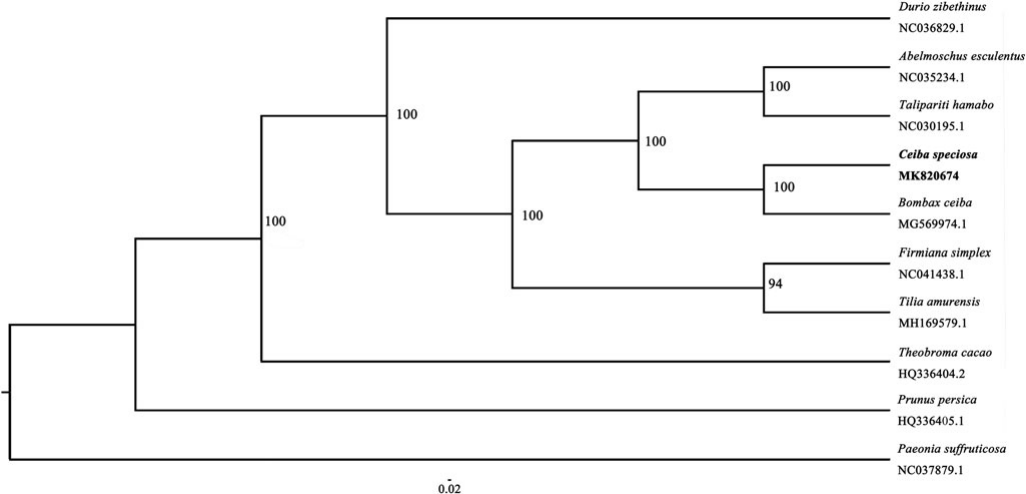
Maximum likelihood tree based on the chloroplast genome sequences of ten species of Malvaceae, Paeoniaceae and Rosaceae, which shows the phylogenetic position of *C. speciosa*. The bootstrap support values were shown next to the nodes; the scale in substitutions per site was shown in the bottom.
